# Gastrocnemius Myocutaneous Flap: A Versatile Option to Cover the Defect of Upper and Middle Third Leg 

**DOI:** 10.29252/wjps.7.3.314

**Published:** 2018-09

**Authors:** Jagdeep Rao, Rakesh Tawar, Rakesh Dawar

**Affiliations:** 1 Department of Plastic Reconstructive and Burns Surgery, SMS Medical College, All India Institute of Medical Sciences, New Delhi, India;; 2 Department of Plastic and Reconstructive Surgery, Sarojini Marg, Jaipur, India

**Keywords:** Gastrocnemius, Leg defect, Myocutaneous flap, Reconstruction, Trauma

## Abstract

**BACKGROUND:**

Large soft tissue leg defect involving upper and middle third remains a therapeutic challenge. The objective of this study was to evaluate the effectiveness and versatility gastrocnemius myocutaneous flap cover for post traumatic large defect of upper and middle third of leg.

**METHODS:**

This prospective study was conducted from January 2015 to January 2017 on 25 consecutive cases of post-traumatic upper and middle third leg defect who were treated with gastrocnemius myocutaneous flap and the functional and aesthetic outcome were evaluated.

**RESULTS:**

There was no case of complete flap failure. Partial skin necrosis occurred in 2 patients (8%). There was no postoperative hematoma while mild discharge was seen in only 4(16%) patients. With regard to the donor site morbidity, no functional deformity was seen in follow up period. The procedure was found to be reliable, technically easy and aesthetically acceptable.

**CONCLUSION:**

Post-traumatic large defects of leg extending in upper and middle third were easily covered with the help of regional gastrocnemius myocutaneous flap with excellent outcome and aesthetically acceptable coverage of skin without any major complications or long term morbidity.

## INTRODUCTION

The repair of defect after severe trauma with extended soft tissue loss over anterior aspect of upper and middle third of leg is challenging because of the paucity of available local options for large defect. Available options like cross leg flap cover and free flap, are both poles apart, with cross leg flap being an age-old method while free flap still lacks available expertise. Despite severe trauma over anterior aspect of leg, the posterior region of leg is usually spared and thus, gastrocnemius myocutaneous flap is an excellent and versatile option available for a large defect because of its reliable anatomy and vigorous blood supply.^1^^,^^2^

Gastrocnemius myocutaneous flap was originally described in 1977,^3^ for providing coverage over the knee region. The gastrocnemius muscle has two heads: medial and lateral. Each head can be mobilized on its own neurovascular pedicle.^4^ The Sural arteries, one medial and one lateral, supply the medial and the lateral head of the gastrocnemius muscle, respectively. Each musculo-cutaneous perforator can supply a large area of skin proximally and also a significant amount distal to muscle belly.^5^

Most of the perforators in the gastrocnemius myocutaneous flap are located 7–18 cm from the popliteal crease.^6^^,^^7^ The lesser saphenous vein can also be included with the flap in order to increase venous drainage or as the sole outflow for the flap.^8^ Use of Doppler to map the location of skin perforators, may aid in ensuring adequate vascularity to the skin.

The aim of this study is to evaluate the use of gastrocnemius myocutaneous flap as a versatile option for the coverage of large post traumatic leg defect.

## MATERIALS AND METHODS

From January 2015 through January 2017, 25 consecutive cases of large post-traumatic defect in upper and middle third of leg were treated with gastrocnemius myocutaneous flap in department of plastic surgery. All cases were initially treated with debridement and, in some cases, fixation of fractured bone with external fixator. Their age ranged from 14 to 56 year. Patients having associated injuries that required multidisciplinary treatment, were excluded from the study. All nonviable and poorly vascularized tissues were aggressively debrided and washed with hydrogen peroxide and then the defect was measured. The procedure was done under regional anesthesia with the patient laid in prone position. According to the defect, skin pedicle was marked over medial or lateral head of gastrocnemius muscle, not crossing more than 1 cm of midline. Midline and popliteal crease were marked. Elevation was similar in both the flaps. Skin incision was given over the previously marked site and extended distally up to tendo-Achilles region. The gastrocnemius muscle was identified and separated from soleus muscle and the attachment with Achilles tendon was divided. Both heads were sharply divided and musculocutaneous flap was raised up to the knee joint with preservation of the vascular pedicle. 

To increase the approach of the flap, we divided the origin of the muscle which also increased the rotation point of the flap. The upward mobility of the flap was ultimately facilitated by dividing the proximal part of the skin which created an island myocutaneous flap. After tunneling, flap was insetted over the defect with sutures. Suction drainage and post-operative splinting was done in all patients. Patients were discharged from the hospital after primary dressing and review in out-patient clinic was done after 15 days. Follow up was done for 4-6 months during which, outcome was evaluated with respect to functional results and patients’ aesthetic satisfaction graded as satisfied, acceptable and not satisfied. Informed consent was obtained from all patients included in the study, whenever applicable. All procedures performed in the studies involving human participants were in accordance with the ethical standards of the institutional and/or National Research Committee and with the 1964 Helsinki declaration and its later amendments or comparable ethical standards.

## RESULTS

This study included 25 cases; out of them 22 were male and 3 were female. Among male patients, in 17, medial muscle head and in 5, lateral muscle head were used, while these figures for females were 2 and 1. All case were admitted in trauma center and treated with debridement and fracture fixations and then residual defect coverage was done. Most patients were of the age group of 25 to 50 years. Table 1 demonstrates age and sex wise distribution of cases. Maximum size of defect that was covered by gastrocnemius myocutaneous flap was 16 cm in length, while most of the defects were of 5-10 cm in size. Mostly (19), the defect was covered proximally by medial head gastrocnemius myocutaneous flap. Table 2 shows the size of the flap used.

**Table 1:** Age and sex wise distribution of cases

**Age (years)**	**Male (n=22)**	**Female (n=3)**	**Total (n=25)**
0-15	02	00	02
16-25	06	01	07
26-50	12	02	14
>50	02	00	02

**Table 2:** The size of the flap

**Size (cm)**	**Medial head**	**Lateral head**	**Total (n=25)**
0-5	04	01	05
5-10	07	02	09
10-15	13	00	13
>15	01	00	01

**Fig. 1:**
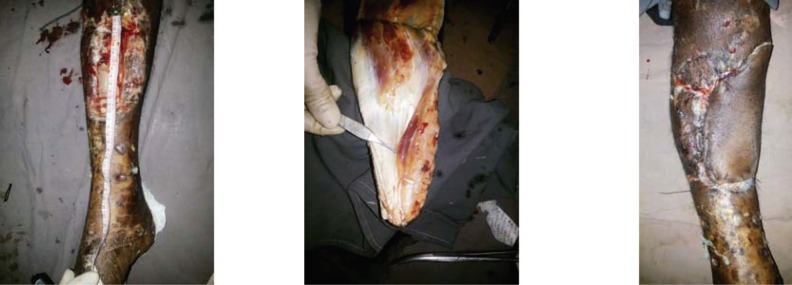
A. Pre-operative picture of a large post traumatic defect of 15 cm size of right leg involving upper and middle third. B. Medial sided gastrocnemius myocutaneous flap raised for the coverage of the defect. C. Post-operative picture showing proper coverage of the defect by gastrocnemius myocutaneous flap

**Fig. 2:**
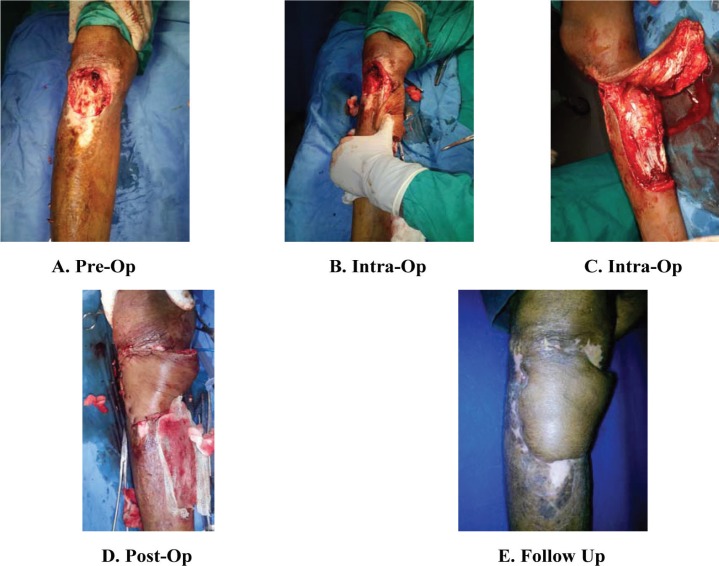
A. Pre-operative picture showing defect in the upper third of right leg along with the unstable area approaching the middle third of leg. B, C. Intra-operative pictures of debridement and raising of medial gastrocnemius myocutaneous flap. D. Post-operative picture after insetting. E. Picture on 15^th^ day, post-operative, showing complete coverage

**Fig. 3:**
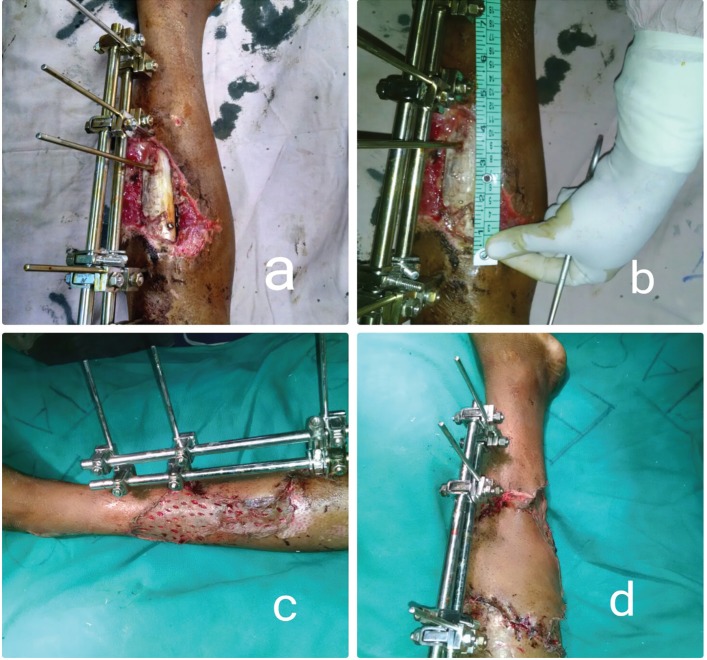
a, b. pre-operative picture of a patient with compound fracture tibia left leg showing external fixator and measurement of defect, i.e., 13 cm long. c. donor area covered with split thickness skin graft. d. Gastrocnemius myocutaneous flap adequately covered the defect

After the flap elevation, donor site closure was achieved in 12 patients, while in remaining patients donor site required split thickness skin grafting. There was no case of complete flap loss. Partial necrosis occurred in 2 patients that were treated with debridement and split thickness skin grafting. No functional deficit of donor site was noted in any of the cases. Discharge from recipient site, post-operatively, was seen in 4 patients and was managed conservatively. Table 3 demonstrates the complications of flap cover. Average duration of hospital stay was 5-7 days. Follow up period ranged from 4 to 6 months during which stable wound coverage by the myocutaneous flap was noted. One patient was lost in follow up. No patient complained of any functional deficit and all patients had an aesthetically acceptable appearance with complete coverage of the defect (Figures 1-3). 

**Table 3:** Complications of flap cover

**Complications**	**Medial head**	**Lateral head**
Functional deficit	00	00
Infection	03	01
Partial flap necrosis	00	02
Complete flap loss	00	00

## DISCUSSION

Skin and soft tissue defect of leg after trauma is commonly encountered for which early and precise management of the patients may improve the overall outcome aesthetically and functionally. There are many options for upper and middle third of leg defect separately, but the large defect involving area between upper and middle third or transition zone between two has very limited options like cross leg flap, gastrocnemius flap and free flap. Cross leg flap is rarely used now since it leads to long term morbidity and discomfort to the patients due their cumbersome position.^9^

In era of microsurgery, free flap is the most commonly employed flap for coverage of any defect, but free flap leads to donor site morbidity and requires expertize. Moreover intensive post-operative monitoring, requirement of healthy recipient vessel and chances of re exploration are the areas of concern with free flap. Use of cross leg flap and free flap for upper and middle third of leg can be obviated by gastrocnemius myocutaneous flap. The flap has a reliable vascular pedicle along with a large skin paddle as per need.^9^

Advantage of gastrocnemius myocutaneous flap is that the flap can cover the defect up to middle third of leg successfully without any complications. In our study, functional loss was not seen in any patient and the contour deformity was acceptable. This result is similar to that found in the study of Kroll *et al. *who have concluded that functional and aesthetic outcome is acceptable when only one flap is raised.^10^ Scar can be avoided by tunneling of the flap beneath skin-bridge.

Bashir *et al.* in their study described gastrocnemius tenocutaneous island flap at the lower end of the muscle.^6^ In our study, using skin paddle, detaching their origin, multiple scoring of the muscle and oblique placement of flap were used to improve the reach of flap. In order to stretch the flap distally, the pedicle was skeletonized and the fascia over the proximal muscle was removed. The rate of complications encountered in our study are comparable to other large studies, with only minor complications seen.^6^^,^^7^^,^^10^ Complete flap necrosis did not occur in any case in our study, although it was seen in the study of Chung *et al.* which they had attributed to inadequate tunneling of flap.^11^

In the era of microsurgery, the gastrocnemius myocutaneous flap for reconstruction of defect involving the upper and middle third of the leg is very attractive and versatile option for plastic surgeon. It is a simple technique allowing rapid, durable and reliable coverage of these defects without sacrificing a nerve or a major vessel to the foot. No donor site morbidity as functional deformity was noted in any patient.

## CONFLICT OF INTEREST

The authors declare no conflict of interest.
